# Delayed surgical repair in double penile fracture: Insights and outcomes: A case report

**DOI:** 10.1016/j.ijscr.2024.109623

**Published:** 2024-04-06

**Authors:** Wael Gazzah, Yassine Najjai, Rayen Lahouar, Mahdy Mzoughi, Zied Mansi, Salem Braiek

**Affiliations:** a University of Sousse, Faculty of Medicine, Department of Urology, Ibn El Jazzar Hospital, Kairouan, Tunisia; b University of Sousse, Faculty of Medicine, Department of Orthopedic Surgery, Ibn El Jazzar Hospital, Kairouan, Tunisia

**Keywords:** Penile fracture, Emergency surgical procedures, Ultrasonography, Erectile dysfunction, Urology

## Abstract

**Introduction and importance:**

Penile fractures, though rare, demand urgent surgical attention due to their potentially severe consequences. This case report illustrates the significance of prompt and comprehensive imaging with surgical exploration in managing a delayed presentation of a double penile fracture.

**Case presentation:**

A 27-year-old male sustained a penile injury during sleep, presenting to our department 36 h post-trauma. His clinical symptoms included significant penile swelling, deviation, and the characteristic ‘eggplant’ deformity. Ultrasonography revealed extensive subcutaneous edema and a substantial hematoma at the penile base, with a disruption in the tunica albuginea. Surgical exploration identified two distinct fractures in the corpora cavernosa, which were successfully repaired. The patient experienced a rapid and complication-free recovery, regaining full erectile function within four days.

**Clinical discussion:**

This case underlines the anatomical complexity of penile fractures. Despite the delay in seeking medical attention, the outcome was favorable, challenging the notion that immediate surgery is crucial for avoiding long-term complications. The literature suggests that delayed surgery might not significantly impact long-term outcomes, especially in the absence of urethral involvement, a perspective supported by our case findings.

**Conclusion:**

Penile fracture requires a nuanced approach to diagnosis and treatment. The case demonstrates that while immediate surgical intervention is ideal, delayed repair can also result in positive outcomes under certain conditions. This report contributes to the growing body of evidence suggesting the potential for re-evaluating current clinical guidelines for penile fracture management.

## Highlights

• The article discusses a rare situation of a patient with two penile fractures.
• The patient had successful surgery even after a 36-h delay.
• Advanced imaging techniques were key to diagnosing and treating the fractures.
• The patient recovered quickly and effectively thanks to the detailed surgical method used.
• This case suggests that medical guidelines for treating penile fractures might need updating.

## 1 Introduction

Penile fracture, a relatively rare but urgent surgical condition, is typically a consequence of trauma to the erect penis, leading to rupture of the tunica albuginea that surrounds the corpora cavernosa. The standard management protocol emphasizes prompt diagnosis and immediate surgical intervention to ensure optimal outcomes, minimizing complications such as erectile dysfunction or penile curvature [1].

Early surgical repair, ideally performed within hours to a few days after injury, is correlated with better functional outcomes and reduced morbidity [2]. However, factors such as societal taboos and cultural barriers often contribute to delays in seeking medical care, leading to late presentations and potentially more complex clinical scenarios [3]. This hesitation and the resultant delay in medical attention can also contribute to an underestimate of the incidence of penile fractures in certain populations.

This case report presents a unique scenario of a young male patient with a delayed presentation of a penile fracture. In particular, this case was distinguished by the presence of two separate fractures in the tunica albuginea, which were effectively repaired without the subsequent complications. The case highlights the importance of meticulous imaging and exploration during surgery. In particular, it underscores the need for complete degloving of the penis shaft, allowing a thorough assessment to identify and address all injuries. This approach is crucial in avoiding potential long-term physical and psychological sequelae. We report the case according to the surgical case reports (SCARE) guidelines [4].

## 2 Case presentation

A 27-year-old male patient presented a history of penile trauma that occurred during sleep. The patient experienced a traumatic event in which he accidentally turned his erect penis, resulting in an audible'snap’ sound and subsequent intense pain. This incident was followed by immediate detumescence and diffuse swelling of the penis. In particular, the patient delayed seeking medical attention and came to our facility approximately 36 h after injury.

**Fig. 1:**
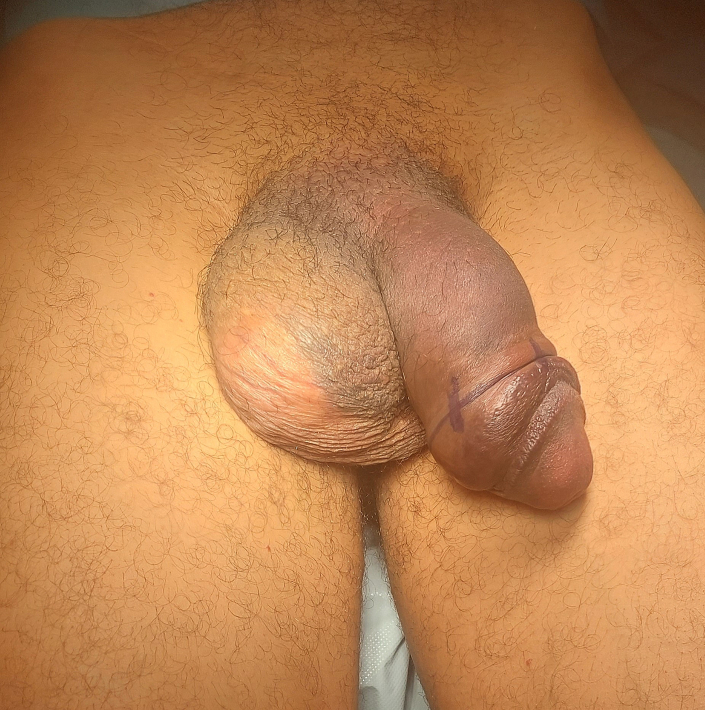
Penile fracture with eggplant deformity.

On clinical examination, significant swelling of the penis and deviation to the right were observed, along with the characteristic eggplant deformity (Fig. 1). There was no evidence of urethral injury. A detailed penile ultrasound revealed extensive subcutaneous edema that extended to the scrotum. Additionally, a large hematoma was identified near the base of the penis, along with a 9 mm disruption in the tunica albuginea of the right corpus cavernosum.

**Fig. 2:**
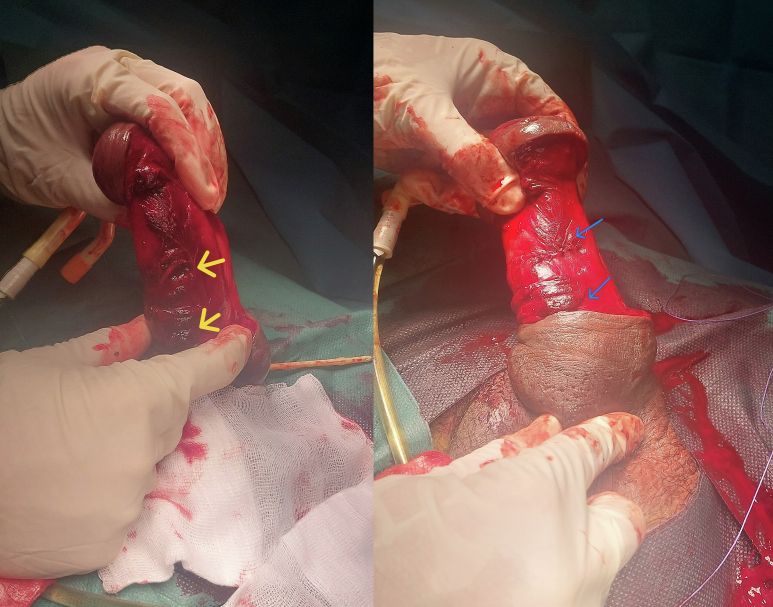
Two visible fractures: before then after repair.

**Fig. 3:**
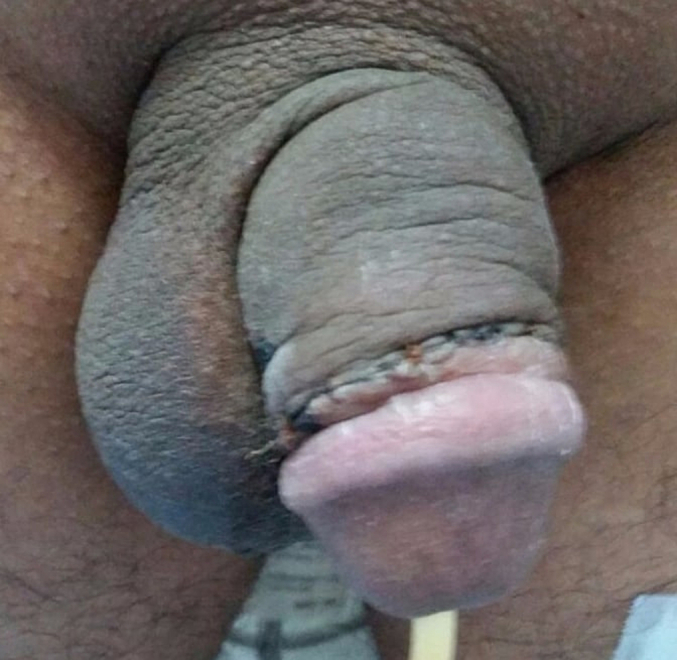
Second postoperative day after repair of penile fractures.

Emergent surgical exploration under spinal anesthesia, during which degloving of the penis revealed two distinct fractures at the base of the corpora cavernosa, measuring 10 and 7 mm, respectively. These lacerations were meticulously repaired using interrupted 3–0 Vicryl sutures (Fig. 2). The postoperative course was uneventful; edema was significantly reduced by the second day and the patient regained full erectile function on the fourth day (Fig. 3).

During a 12-month follow-up period, the patient has not reported complications.

## 3 Discussion

The penile shaft, which comprises the two dorsolateral corpora cavernosa and a ventral corpus spongiosum, each encased in a tunica albuginea, is a complex anatomical structure. The glans penis, an extension of the corpus spongiosum, incorporates the anterior urethra. The surrounding structures play a crucial role in the structural integrity of the penis [5].

Penile fractures in the erect state, though rare, are recognized as urgent urological emergencies. These injuries are predominantly the result of trauma during sexual activity, with vaginal intercourse being cited as the most common cause. However, other mechanisms have been identified, including masturbation, accidental rolling during sleep, and even self-manipulation [6,7]. Risk factors exacerbating the probability of such fractures include the use of pharmaceutical agents that prolong erection, existing urethral or periurethral infections, and previous penile trauma [8]. The underlying mechanism involves a sudden increase in intracavernosal pressure against the thinned tunica albuginea during erection, which typically reduces in thickness from approximately 2.4 mm in its flaccid state to just 0.25–0.5 mm when engorged [9].

According to the extensive review by Eke that included 1331 cases in 183 publications, the majority of cases of penile fracture are reported in the Mediterranean region, mainly affecting individuals in their 30s and 40s [3]. The classic clinical presentation includes acute penile pain, loss of erection, swelling, and deviation, commonly accompanied by urinary problems. The predominant medical consensus advocates for prompt surgical intervention, using absorbable sutures to repair the tunica albuginea, as this approach significantly reduces the risk of long-term complications such as erectile dysfunction, penile curvature, and urethral fistulae [3].

The diagnosis of penile fracture is based primarily on clinical evaluation, supplemented by various imaging techniques to confirm the diagnosis and delineate the extent and location of the injury. These modalities include cavernosography, retrograde urethrography, magnetic resonance imaging (MRI) and Doppler color ultrasound [5]. Each has its merits and limitations; for example, cavernosography, while accurate, is invasive and involves radiation exposure, and hence its less frequent use. Retrograde urethrography is reserved mainly for cases with suspected urethral injuries. Magnetic resonance imaging offers superior evaluation with its multiplanar capabilities and excellent tissue contrast, but its high cost and limited accessibility are notable drawbacks [5].

In our practice, high-frequency sonography (7.5 MHz–12.0 MHz) is preferred for assessing penile trauma. This noninvasive technique allows for detailed examination of structures typically smaller than 1 mm and evaluates penile vascularity [10]. Sonography effectively located the tear as a disturbance in the echogenic tunica albuginea and identified the accompanying hematoma in this case. Additionally, this modality can detect small tunica tears and, through color Doppler, reveal leaks from the cavernosal bodies by applying pressure to the penile shaft. Although ultrasound can suggest a rupture of the urethral wall, retrograde urethrography may still be necessary to confirm such injuries.

Our case is particularly notable for the presentation of two distinct fractures within the corpus cavernosum, located approximately 3 cm apart, a rarity in the current literature where single fractures are reported more frequently [11]. The identification of a secondary fracture site in this case reinforces the importance of meticulous surgical exploration. This comprehensive approach is essential to detect all injuries and prevent possible suboptimal outcomes, highlighting the need for a detailed intraoperative evaluation during degloving of the penile shaft.

Although the consensus in the literature and clinical practice strongly supports urgent surgical intervention for penile fractures, our case adds a new dimension to the debate on the timing of surgery. It presents an unusual scenario in which surgical repair was performed more than 36 h after trauma due to delayed presentation, but resulted in a positive outcome. This challenges the traditional urgency paradigm and suggests that in certain cases, particularly those without urethral involvement, delayed surgery may not adversely affect long-term outcomes such as erectile function and penile deformity. This notion is somewhat supported by studies such as those of Kozacioglu et al., which found satisfactory results with surgeries performed on average 11.3 ± 8.5 h after injury [12,13]. However, it is crucial to note that our case significantly extends this window, demonstrating a successful outcome in a scenario with a much longer delay.

This case report contributes to the ongoing discussion about the management of penile fractures, particularly regarding surgical timing. It illustrates that delayed surgery, under certain conditions, can still lead to favorable outcomes, challenging existing paradigms, and underscoring the need for more research. Such insights are crucial in developing more flexible and patient-centered approaches to the treatment of this rare but significant urological emergency. Our findings also suggest the potential for the revision of current guidelines to accommodate a wider range of clinical scenarios.

## 4 Conclusions

Penile fracture, although rare, represents an urgent surgical condition that requires prompt and effective management. Traditionally, the diagnosis of this condition is made primarily through clinical evaluation, but, as illustrated in our case, high-resolution sonography and color Doppler imaging are invaluable for accurately identifying the location and severity of the injury. These imaging techniques offer a more precise and non-invasive approach, enhancing the diagnostic process.

This case report highlights several critical aspects of penile fracture management. Firstly, it emphasizes the need for a complete degloving of the penis during surgery. This approach is crucial to thoroughly identify and repair all fracture sites, as multiple fractures, such as those observed in this case, can otherwise be overlooked. The success of the surgical procedure in our report, which involved repairing two separate fractures, demonstrates the importance of meticulous surgical exploration.

Second, our case challenges the prevailing paradigm that emphasizes immediate surgery for penile fractures. We present a scenario where delayed surgical intervention, performed more than 36 h after injury, resulted in positive patient outcomes. This finding is particularly significant, as it suggests that in certain circumstances, particularly in the absence of urethral injury, delayed surgery may not adversely affect long-term results, including erectile function and penile morphology.

However, it is crucial to acknowledge that our case represents an individual scenario and generalizations should be made with caution. Future research involving larger cohorts and comparative studies between early and delayed surgical interventions is imperative. Such studies are essential to definitively determine whether the timing of surgery significantly impacts outcomes. The insights gained from this research could lead to a potential re-evaluation and update of current clinical guidelines, fostering more flexible and patient-tailored approaches in the management of penile fractures.

## Patient perspective

The patient expressed relief and satisfaction with the treatments received, highlighting their effectiveness and the care provided by the medical team.

## Informed consent

Written informed consent was obtained from the patient for the publication of this case report and any accompanying images. A copy of the written consent form is available for review by the Editor-in-Chief of this journal upon request.

## Ethical approval

This study is exempt from ethical approval as per the policies of Ibn El Jazzar Hospital.

## Funding

No funding was received for conducting this study.

## Author contribution

All authors have contributed equally to the work reported in this manuscript, including the conception, design, execution, data acquisition, analysis and interpretation, and the drafting and revising of the manuscript for important intellectual content.

## Guarantor

Wael Gazzah.

## Research registration number

RSN 00345/2024.

## Conflict of interest statement

The authors declare that they have no conflicts of interest concerning this article.
